# Direct effects of HP Acthar Gel^®^ on human B lymphocyte activation in vitro

**DOI:** 10.1186/s13075-015-0823-y

**Published:** 2015-10-27

**Authors:** Nancy J. Olsen, Dima A. Decker, Paul Higgins, Patrice M. Becker, Carl A. McAloose, Ann L. Benko, William J. Kovacs

**Affiliations:** Division of Rheumatology, The Pennsylvania State University, College of Medicine, 500 University Drive, Hershey, 17033 PA USA; Autoimmune and Rare Diseases Business, Mallinckrodt Pharmaceuticals, 6011 University Boulevard, Ellicott City, 21043 MD USA; Division of Endocrinology, Diabetes, and Metabolism, The Pennsylvania State University, College of Medicine, 500 University Drive, Hershey, PA 17033 USA

**Keywords:** Adrenocorticotropic hormone, ACTH, B lymphocytes, Humoral immunity, Immunoglobulins

## Abstract

**Introduction:**

Both clinical experience and experimental evidence have suggested that Adrenocorticotropic hormone (ACTH) might directly exert immunomodulatory effects not dependent on adrenal steroidogenesis.

**Methods:**

The direct effects of H.P. Acthar Gel® (Acthar), a repository preparation containing a porcine ACTH analogue, on human B lymphocyte function were studied in vitro using peripheral blood B cells isolated using anti-CD19 coated magnetic beads and activated by interleukin 4 (IL-4) and CD40 ligand (CD40L). Analysis of expression of messenger RNA (mRNA) encoding activation-induced cytidine deaminase (AICDA) was carried out by quantitative real-time polymerase chain reaction (PCR). Cellular proliferation was assessed by a flow cytometric technique using intracellular staining with carboxyfluorescein succinimidyl ester (CFSE). Immunoglobulin G (IgG) production was measured in cell supernatants using an immunoassay.

**Results:**

Acthar was found to exert acute, dose-dependent inhibitory effects on IL-4/CD40L–mediated induction of the expression of activation-induced cytidine deaminase (AICDA) after 24 hours, as well as sustained inhibition of B cell proliferation and IgG production during five more days of culture, without deleterious effects on B cell viability.

**Conclusions:**

These experiments demonstrate that Acthar can exert direct effects on the humoral immune system independent of any role in the regulation of adrenal steroidogenesis. Although the impact of these findings on clinical disease was not evaluated in this study, these data support the therapeutic potential of Acthar for the management of autoimmune diseases characterized by B cell activation and aberrant humoral immune function.

## Introduction

Adrenocorticotropic hormone (ACTH), also known as corticotropin, is a member of a group of peptide hormones (the melanocortins) derived from the proopiomelanocortin (POMC) gene by cell-specific transcription patterns and post-translational processing [1]. The principal source of these gene products is the pituitary and central nervous system, although POMC gene expression occurs at lower levels in a variety of tissues [2]. The melanocortins exert their physiologic effects via binding to one or more of the members of a family of specific G-protein coupled melanocortin receptors (MCRs) and activation of the Jak/STAT pathway [3, 4].

While ACTH exerts its principal physiologic effect to stimulate adrenal growth and to acutely regulate glucocorticoid production by the cells of the adrenal cortex [1], extra-adrenal targets of ACTH action were postulated over 50 years ago [5] and the recognition that MCRs are expressed on a wide variety of cell types, including cells of the immune system, has stimulated renewed interest in the immunomodulatory properties of these peptides [6]. Radioligand binding studies on separated populations of rat lymphoid cells demonstrated the highest levels of specific ACTH binding on B lymphocytes, while T lymphocytes exhibited about half that receptor capacity and thymocytes had low or undetectable levels of ACTH surface binding [7]. At the mRNA level, quantitative PCR experiments have shown lower levels of MCR gene expression in human peripheral B cells compared with CD4^+^ T cells, but mRNAs for MCR1, MCR3, and MCR5 (all of which bind ACTH) have been identified in the CD19^+^ cell population [8].

ACTH may exert effects on physiologic processes either directly (via MCR-mediated action on target cells) or indirectly, through stimulation of adrenal steroidogenesis. Early studies by both Hench et al. [9] and Thorn and Bayles [10] compared the effects of ACTH with those of glucocorticoids on the clinical features of human autoimmune diseases such as rheumatoid arthritis. Both groups concluded that an intact adrenal corticosteroid biosynthetic response was essential for ACTH to mimic the clinical effect of exogenous glucocorticoid therapy [10, 11]. Until it became technically possible to examine hormone action in isolated cells in culture, it was believed that ACTH effects on immune system function could only be indirectly exerted by stimulation of endogenous glucocorticoid production by the adrenals. The first of such studies in vitro reported inhibitory activity of ACTH on peripheral B-lymphocyte function [12]. In that study, ACTH preparations from porcine pituitary glands were found to suppress antibody production by murine spleen cells in vitro in response to both T-cell-dependent and T-cell-independent antigens that had been used as immunogens in vivo. The observed suppressive effects of ACTH occurred at hormone concentrations estimated to be in the micromolar range [12]. Subsequent studies suggested contradictory effects of ACTH on human tonsillar B cells activated in vitro by interleukin (IL)-2 or IL-4, reporting enhanced cellular proliferation and antibody production [13, 14]. Proinflammatory activities in T cells and natural killer cells have also been described [15, 16]. Other investigators have reported suppressive effects of synthetic ACTH on immunoglobulin E production in vitro by cultures of human peripheral blood mononuclear cells but such effects were not observed in highly purified B-cell populations [17].

We tested the effects of H.P. Acthar Gel® (Acthar, Mallinckrodt Pharmaceuticals, Ellicott City, MD, USA), a porcine pituitary-derived ACTH analog approved for the treatment of certain autoimmune disorders in which B-cell dysfunction may contribute to disease pathogenesis. Porcine ACTH differs from human ACTH at a single amino acid (position 31) [18] but no differences have been identified in the steroidogenic, melanotrophic, or lipolytic activities of the ACTH peptides from these two species [19]. We examined the effects of this clinically utilized preparation on human B-lymphocyte function in vitro, using highly purified B-cell populations cultured in the absence of glucocorticoids and stimulated by recombinant IL-4 and CD40 ligand (CD40L) as specific B-cell activating signals.

## Methods

### Human subjects, B-lymphocyte preparation, and culture

Healthy volunteer subjects were recruited for the study and gave informed consent to participate in the protocol approved by the Penn State/Milton S. Hershey Medical Center Institutional Review Board. The volunteers included 16 females and nine males, with overall mean (± standard error of the mean (SEM)) age of 37 (±2) years; each experiment was carried out with a unique individual donor. Peripheral blood mononuclear cells were prepared by density gradient centrifugation on Histopaque 1077 (MP Biomedicals, Santa Ana, CA, USA), and peripheral B lymphocytes were then isolated using magnetic CD19 MicroBeads (Miltenyi Biotec, San Diego, CA, USA) and a MidiMACS Separator (Miltenyi Biotec). B cells prepared using this method were analyzed by flow cytometry after staining with anti-CD20 antibodies and found to be >90 % pure. Recovered B cells were resuspended in complete medium (RPMI 1640 with no phenol red, supplemented with 9 % charcoal-stripped fetal bovine serum, 1 % GlutaMAX-1, and 100 I.U./ml penicillin/100 μg/ml streptomycin). Stimulated cultures (using 10 ng/ml IL-4 and 2 μg/ml recombinant human CD40L from HEK293 cells; both R&D Systems, Minneapolis, MN, USA) and control cultures were plated at a density of 0.5 × 10^6^ –1.0 × 10^6^ cells/ml. Acthar or placebo (provided in blinded fashion; Mallinckrodt Pharmaceuticals, St. Louis, MO, USA) was added to cultures to test the effects of varying estimated concentrations of the active compound. Test compounds were freshly prepared in complete medium on the day of the experiment, within 1 hour prior to addition to cell cultures. Placebo was identical to Acthar, except that it did not contain any active compound. Both the Acthar and the placebo preparations were identical to those used in both clinical studies [20] and animal studies in vivo [21]. Cells were incubated at 37 °C with 5 % CO_2_ for varying lengths of time from overnight to 6 days. No additions were subsequently made to cultures during the incubation period.

### Assays for immunoglobulins in cell culture supernatants

Immunoglobulin G (IgG) and immunoglobulin M (IgM) levels in cell culture supernatants were measured using immunoglobulin heavy chain-specific (IgG) and intact molecule-specific (IgM) microsphere agglutination assays (Pierce Easy Titer; Thermo-Fisher, Rockford, IL, USA).

### Quantitation of cellular proliferation in culture

To assess effects of test compounds on B-cell proliferation, cells were stained with carboxyfluorescein succinimidyl ester (CFSE; CellTrace from Molecular Probes, Eugene, OR, USA) using the manufacturer’s protocol prior to culture and stimulation in vitro. After completion of the incubation period, cells were harvested, washed, and analyzed by flow cytometry using a BD FACS Canto II instrument (BD Biosciences, Franklin Lakes, NJ, USA) in the Penn State Hershey Flow Cytometry Core Facility. The distribution of peaks of fluorescence intensity was analyzed using FlowJo software (TreeStar, Ashland, OR, USA) to yield the parameters of percentage of cells that divided, division index (the average number of divisions of all cells), expansion index (the fold-expansion of all cells), proliferation index (the average number of divisions of a responding cell), and replication index (the fold-expansion of responding cells).

### Flow cytometric assessment of cell viability

Cultured B cells were assessed for viability using the LIVE/DEAD Fixable Dead Cell Stain kit with green fluorescent reactive dye (Molecular Probes). Cells were harvested from culture plates at the end of various treatments, pelleted by centrifugation at 300 × *g* for 10 minutes, washed, and resuspended in phosphate-buffered saline (PBS). Cells were stained with green fluorescent amine reactive dye reconstituted in dimethylsulfoxide (DMSO; 1 μl per million cells), stored on ice in the dark for 30 minutes, and then washed once with PBS. Cells were analyzed for dye exclusion (live cells) or inclusion (dead cells) by flow cytometry on a BD FACS Canto II instrument in the Penn State Hershey Flow Cytometry Core Facility using the 488 nm excitation laser and 530 nm detection band. Parallel samples were analyzed by visual microscopic inspection for trypan blue dye exclusion.

### RNA isolation and RT-PCR quantitation of activation-induced cytidine deaminase

RNA was isolated from harvested cells using the RNeasy Mini Kit (Qiagen, Germantown, MD, USA) according to the manufacturer’s instructions. The RNA concentration was quantitated using a Nanodrop 2000c spectrophotometer (Thermo Fisher Scientific - Nanodrop Products, Wilmington, DE, USA) . Reverse transcription of RNA was performed using the High Capacity RNA to cDNA Kit (Applied Biosystems, Life Technologies Corporation, Carlsbad, CA, USA).

Activation-induced cytidine deaminase (AICDA) mRNA expression was quantitated using real-time PCR technique with Taqman® reagents from Applied Biosystems (Gene Expression Assay Hs 00221068), with GAPDH (Gene Expression Assay Hs 03929097) as a control mRNA for calculation of induction using the ΔΔCt method. Assays were run on an Applied Biosystems Quant Studio 12 K Flex real-time PCR system in the Penn State Hershey Genome Sciences facility.

### Statistical analyses

Statistical analyses were carried out using Prism software (version 6.0; Graph Pad, San Diego, CA, USA) or SAS (SAS Institute Inc., Cary, NC, USA) Results are presented as mean and SEM values. Multiple measurements were analyzed for statistical significance using analysis of variance (ANOVA) with Tukey’s multiple comparison post test or with the Kruskal–Wallis test with Dunn’s multiple comparison post test for experiments in which datasets were not normally distributed. *p* <0.05 was considered significant.

## Results

**Fig 1:**
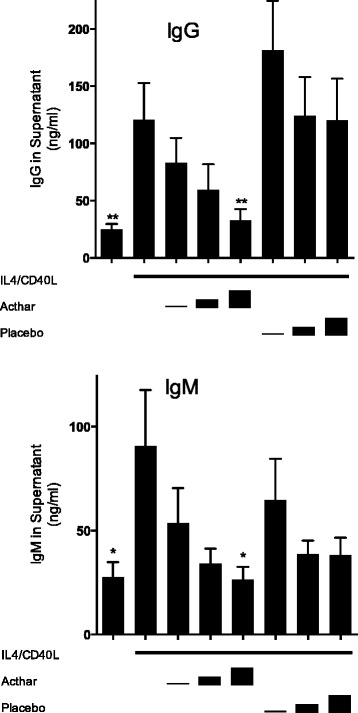
Immunoglobulin production by activated human B lymphocytes in vitro is suppressed by Acthar. IgG (*top panel*) and IgM (*bottom panel*) were measured in supernatants from human peripheral B cells cultured for 6 days under basal conditions, with added IL-4/CD40L alone, or with IL-4/CD40L plus added Acthar at ACTH analog concentrations of approximately 0.124, 1.24, or 2.49 μM (*stepped bars*). Parallel cultures contained IL-4/CD40L plus matched volumes of placebo. Supernatants were harvested and assayed for IgG or IgM concentration and results analyzed by ANOVA (Kruskal–Wallis test, *p* <0.0001 for IgG; *p* = 0.0551 for IgM). **Significantly different from IL-4/CD40L-stimulated samples by Dunn’s post test at *p* <0.01. *Significantly different from IL-4/CD40L-stimulated samples by Dunn’s post test at *p* <0.05. Results shown are mean ± SEM for eight independent experiments. *Acthar* H.P. Acthar Gel®, *CD40L* CD40 ligand, *Ig* immunoglobulin, *IL* interleukin

Treatment of IL-4/CD40L-activated human B cells in vitro with Acthar resulted in a dose-dependent reduction of IgG accumulation in culture supernatants after 6 days (Fig. 1, top panel). Maximal inhibition of IgG production (72.7 %) was noted at an estimated ACTH analog concentration of approximately 2.49 μM (*p* <0.01). Treatment with placebo at identical volumes did not alter IgG levels compared with IL-4/CD40L-stimulated controls. Effects of the Acthar on IgM accumulation in culture supernatants were also observed—with maximal suppression of 70.8 % at the highest concentration of Acthar (*p* <0.05; Fig. 1, bottom panel). Treatment with the placebo preparation did not result in significant effects on IgM.

**Fig 2:**
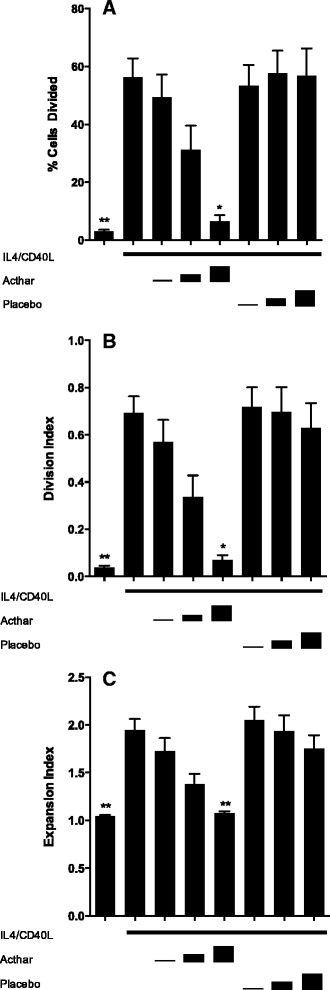
Proliferation of activated human B lymphocytes in vitro is suppressed by Acthar. **a** The percentage of cells that divided, **b** the average number of divisions of all cells (division index), and **c** the fold-expansion of all cells (expansion index) were assessed in CSFE-loaded human peripheral B cells cultured for 6 days under basal conditions, with added IL-4/CD40L alone, or with IL-4/CD40L plus added Acthar at ACTH analog concentrations of approximately 0.124, 1.24, or 2.49 μM. Parallel cultures contained IL-4/CD40L plus matched volumes of placebo. Cells were examined by flow cytometry and data analyzed by ANOVA (Kruskal–Wallis test, *p* <0.0001 for percentage of cells divided, *p* <0.0001 for division index, and *p* <0.0001 for expansion index). **Significantly different from IL-4/CD40L-stimulated samples by Dunn’s post test at *p* <0.01. *Significantly different from IL-4/CD40L-stimulated samples at *p* <0.05. Results shown are mean ± SEM for six independent experiments. *Acthar* H.P. Acthar Gel®, *CD40L* CD40 ligand, *IL* interleukin

During B-lymphocyte activation, the process of immunoglobulin class switching is known to be dependent on cellular proliferation [22]. We therefore examined whether Acthar altered IL-4/CD40L-induced proliferation of human B cells in vitro using CFSE staining to define successive waves of cellular proliferation in culture. We noted a dose-dependent effect of Acthar to inhibit proliferation of activated B cells—with significant reductions in the percentage of cells that divided in culture (Fig. 2a), the average number of divisions of all cells (Fig. 2b), and the fold-expansion of all cells (Fig. 2c). Placebo treatment did not significantly alter any of these parameters of cell proliferation. No significant differences were observed between unstimulated and IL-4/CD40L-stimulated cells with respect to either the number of divisions undertaken by responding cells (“proliferation index”; not shown) or the fold-expansion of responding cells (“replication index”; not shown).

**Fig. 3:**
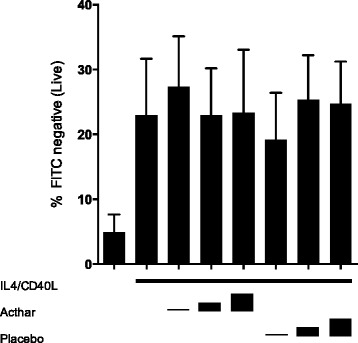
Viability of B lymphocytes in vitro is unaffected by Acthar. The percentage of live cells (negative for staining) was assessed in human peripheral B cells cultured for 6 days under basal conditions, with added IL-4/CD40L alone, or with IL-4/CD40L plus added Acthar at ACTH analog concentrations of approximately 0.124, 1.24, or 2.49 μM. Parallel cultures contained IL-4/CD40L plus volumes of placebo at equal volumes. At the end of the culture period cells were stained with Live/Dead stain as described in Methods. Cells were analyzed by flow cytometry. Statistical analysis (ANOVA) revealed none of the treatment values to be significantly different from IL-4/CD40L-stimulated sample. Results shown are mean ± SEM for six independent experiments. *Acthar* H.P. Acthar Gel®, *CD40L* CD40 ligand, FITC fluorescein isothiocyanate, *IL* interleukin

The effects of Acthar on cell proliferation were not explained by toxic or lytic effects on human B cells. Neither intracellular staining with Live/Dead fluorescent amine reactive dye (Fig. 3) or trypan blue exclusion (not shown) revealed any significant effect of Acthar on these assessments of cell integrity.

**Fig. 4:**
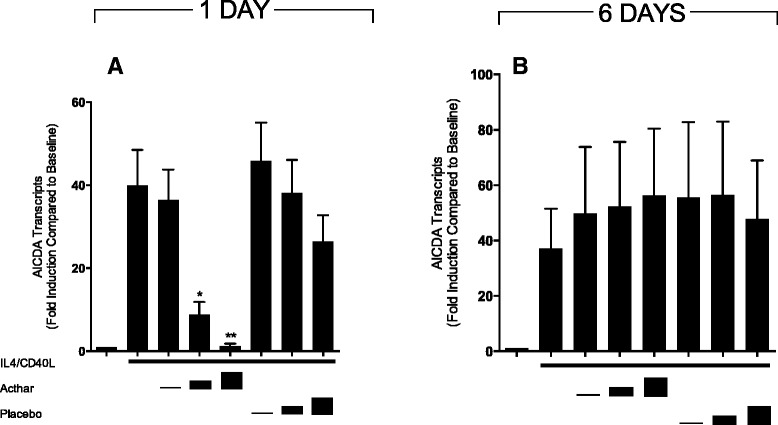
Expression of AICDA mRNA in activated human B lymphocytes in vitro is suppressed by Acthar. Expression of AICDA mRNA was assessed in human peripheral B cells cultured **a** for 1 day or **b** for 6 days under basal conditions, with added IL-4/CD40L alone, or with IL-4/CD40L plus added Acthar at ACTH analog concentrations of approximately 0.124, 1.24, or 2.49 μM. Parallel cultures contained IL-4/CD40L plus placebo at matched volumes. mRNA was isolated and quantitated by RT/PCR as described in Methods. Data were analyzed by ANOVA (*p* = 0.0002 on day 1 and *p* = 0.9973 on day 6). *Significantly different from IL-4/CD40L-stimulated samples by Tukey’s post test at *p* <0.05; **Significantly different from IL-4/CD40L-stimulated samples by Tukey’s post test at *p* <0.01. Results shown are mean ± SEM of six independent experiments at 1 day and of seven independent experiments at 6 days. *Acthar* H.P. Acthar Gel®, *AICDA* activation-induced cytidine deaminase, *CD40L* CD40 ligand, *IL* interleukin

The ability of B cells to switch the class of immunoglobulin produced from IgM to IgG is dependent not only on cellular proliferation, but also on the action of the DNA-modifying enzyme AICDA that initiates the genomic changes resulting in immunoglobulin heavy chain gene class switch recombination as well as somatic hypermutation (SHM) of the variable regions of immunoglobulin heavy and light chain genes [23, 24]. Examination of AICDA expression in IL-4/CD40L-activated human B cells revealed an acute, concentration-dependent inhibitory action of the ACTH analog, reducing the levels of AICDA ordinarily seen in the first 24 hours after such stimulation. At the highest concentration of Acthar, AICDA expression was suppressed on average to 2.67 ± 0.92 % of IL-4/CD40L-stimulated values (*p* <0.05), comparable with the level of expression seen in unstimulated cells (Fig. 4a). After 6 days in culture, AICDA mRNA levels in Acthar-treated cells were comparable with those observed in placebo-treated control cells, consistent with the prediction that active peptide would not still be present in cultures at this time point (Fig. 4b). Placebo-treated cells did not show statistically significant changes in AICDA expression at either time point. These data confirm that Acthar exerted an inhibitory effect on the expression of the key regulator of class switch recombination and SHM in activated human B cells, and that this inhibitory effect is exerted promptly during early phases of exposure to the peptide.

## Discussion

Acthar has US Food and Drug Administration approval for treatment of a number of autoimmune conditions, including the treatment of acute exacerbations of multiple sclerosis, during an exacerbation or as maintenance therapy in selected cases of systemic lupus erythematosus, dermatomyositis, and polymyositis, and as adjunctive therapy for short-term administration in psoriatic arthritis, rheumatoid arthritis, and ankylosing spondylitis (Acthar prescribing information). The therapeutic effects of ACTH preparations in human autoimmune disorders have historically been believed to be the consequence of the stimulatory effect of the hormone on adrenal cortisol production and secretion [10, 11, 25]. However, clinical observation of therapeutic effects of Acthar in glucocorticoid-resistant patients with multiple sclerosis [26], prior in vitro observations of direct effects of ACTH preparations on cultured human B cells [13], and the recognition that human monocytes, macrophages, and T and B lymphocytes express specific MCRs [6, 8, 27] have all suggested that some extra-adrenal effects of this corticotropin preparation might be immunomodulatory. While we have found previously that glucocorticoids acutely downregulate AICDA expression in cultured human B cells [28], the present reexamination of the effects of Acthar on purified human B lymphocytes supports direct effects of a clinically utilized corticotropin preparation on the processes of B-cell activation and immunoglobulin G production, since our experiments were performed in vitro under glucocorticoid-free conditions.

The experiments presented here revealed an acute effect of Acthar to suppress AICDA mRNA expression during the first 24 hours of exposure to IL-4 and CD40L stimulation. As we observed previously with glucocorticoid exposure of human B cells, this effect on AICDA mRNA expression was not sustained, but with Acthar we also found significant suppression of cellular proliferation and IgG production observable 5 days later, an effect not seen with glucocorticoid treatment (glucocorticoid data not shown). Since cellular proliferation is required for initiation of IgG production in activated B cells [22], this effect could be interpreted as antiproliferative. The sustained effects of Acthar exposure on cellular proliferation and IgG production, however mediated, do seem likely to depend on early events, since it seems unlikely that active corticotropin or related peptides remained in the cultures for any sustained period of time. Whether our observations could be the consequence of antagonism of IL-4 action, CD40L action, or other processes operative in B-cell activation remains to be explored.

Recently published studies have identified suppressive effects of Acthar on humoral autoimmunity in the NZB/W F1 mouse model of lupus [21]. The in vivo treatment paradigm utilized in that study included glucocorticoid-treated control animals, but did not explicitly address the question of extra-adrenal versus adrenal-dependent actions of the hormonal preparation (all animal subjects had intact adrenals), so that corticotropin-stimulated production of dehydroepiandrosterone (DHEA) or other potentially immunomodulatory adrenal steroid products could not be formally excluded. The direct effects of Acthar observed on human B cells in vitro in the present work support the contention that adrenal-independent actions directed at B cells themselves were operative in vivo*.* The in vivo treatment of NZB/W F1 mice with Acthar was associated with expanded populations of immature cells in transitional type 1 and marginal zone B cells in the spleen, reductions in germinal center cells, and reductions in circulating IgG and IgM autoantibody titers [21]. The present studies in human cells in vitro corroborate this finding of inhibitory effects of Acthar on immunoglobulin production by circulating B cells that have passed tolerance checkpoints.

Other melanocortin peptides have previously been found to modulate immune function in models of inflammatory bowel disease [29, 30] and in models of T-cell-mediated diseases including autoimmune uveitis [31, 32] and experimental autoimmune encephalomyelitis [33]. Recent studies of melanocortin peptide effects on murine models of systemic lupus erythematosus [21, 34] have now also revealed potential therapeutic roles for these peptides in B-cell-mediated autoimmunity. Our current studies in human cells, cultured in vitro under glucocorticoid-free conditions, suggest that direct effects of one or more components of the Acthar preparation on B lymphocytes may contribute to the disease modulation observed in such models in vivo.

While clearly independent of ACTH effects on glucocorticoid biosynthesis, the molecular mechanisms underlying the observed effects of Acthar on B-lymphocyte function remain to be explored. B lymphocytes have been reported to express MCRs that could mediate these hormonal effects. Human B cells have been found to express MCR1 and MCR3 mRNA [27] and a mouse pro-B cell line has been reported to express MCR5 [4]. Intact corticotropin (as well as alpha MSH, which corresponds to the first 13 amino acids of ACTH) binds to these receptors [6, 27]. Our present studies have not identified the molecular target or the receptor-signaling pathway by which the observed effects on B cells are exerted.

Although it is difficult to directly extrapolate clinically effective dose ranges from in vitro studies, these findings of direct Acthar effects on the function of activated human B lymphocytes have implications for the treatment of autoimmune diseases characterized by B-cell activation and humoral autoimmunity. Abnormalities of the hypothalamic–pituitary–adrenal axis are associated with many autoimmune disorders, and glucocorticoid resistance of immune tissues in many of these patients has been also described [35–37]. The observed direct effects of Acthar on cellular targets in the humoral immune system might thus be exploitable as an additional therapeutic approach.

## Conclusions

These experiments are the first to demonstrate that a clinically utilized ACTH preparation (H.P. Acthar® gel) exerts direct immunomodulatory effects on human B cells activated in vitro. The findings suggest potential for such agents in the treatment of autoimmune diseases characterized by abnormal B-cell activation.

## Abbreviations

AICDA: Activation-induced cytidine deaminase
ACTH: Adrenocorticotropic hormone
Acthar: H.P. Acthar Gel®
ANOVA: Analysis of variance
CD40L: CD40 ligand
CFSE: Carboxyfluorescein succinimidyl ester
DMSO: Dimethylsulfoxide
IgG: Immunoglobulin G
IgM: Immunoglobulin M
IL: Interleukin
MCR: Melanocortin receptor
NK: Natural killer
PBS: Phosphate-buffered saline
POMC: Proopiomelanocortin
SEM: standard error of the mean
SHM: Somatic hypermutation

## References

[1] Kovacs WJ, Orth DN (1998). The adrenal cortex.

[2] Gantz I, Fong TM (2003). The melanocortin system. Am J Physiol Endocrinol Metab..

[3] Mountjoy KG, Robbins LS, Mortrud MT, Cone RD (1992). The cloning of a family of genes that encode the melanocortin receptors. Science..

[4] Buggy JJ (1998). Binding of alpha-melanocyte-stimulating hormone to its G-protein-coupled receptor on B-lymphocytes activates the Jak/STAT pathway. Biochem J..

[5] Evans HM, Sparks LL, Dixon JS, Harris GW, Donovan BT (1966). The physiology and chemistry of adrenocorticotrophin. The pituitary gland.

[6] Ahmed TJ, Montero-Melendez T, Perretti M, Pitzalis C (2013). Curbing Inflammation through endogenous pathways: focus on melanocortin peptides. Int J Inflam..

[7] Clarke BL, Bost KL (1989). Differential expression of functional adrenocorticotropic hormone receptors by subpopulations of lymphocytes. J Immunol..

[8] Andersen GN, Hägglund M, Nagaeva O, Frängsmyr L, Petrovska R, Mincheva-Nilsson L (2005). Quantitative measurement of the levels of melanocortin receptor subtype 1, 2, 3 and 5 and pro-opio-melanocortin peptide gene expression in subsets of human peripheral blood leucocytes. Scand J Immunol..

[9] Hench PS, Kendall EC, Slocumb CH, Polley HF (1950). Effects of cortisone acetate and pituitary ACTH on rheumatoid arthritis, rheumatic fever and certain other conditions. Arch Intern Med..

[10] Thorn GW, Bayles TB (1949). Studies on the relation of pituitary–adrenal function to rheumatic disease. N Engl J Med..

[11] Hench PS, Slocumb CH, Polley HF, Kendall EC (1950). Effect of cortisone and pituitary adrenocorticotropic hormone (ACTH) on rheumatic diseases. J Am Med Assoc..

[12] Johnson HM, Smith EM, Torres BA, Blalock JE (1982). Regulation of the in vitro antibody response by neuroendocrine hormones. Proc Natl Acad Sci U S A..

[13] Alvarez-Mon M, Kehrl JH, Fauci AS (1985). A potential role for adrenocorticotropin in regulating human B lymphocyte functions. J Immunol..

[14] Brooks KH (1990). Adrenocorticotropin (ACTH) functions as a late-acting B cell growth factor and synergizes with interleukin 5. J Mol Cell Immunol..

[15] Kavelaars A, Ballieux RE, Heijnen C (1988). Modulation of the immune response by proopiomelanocortin derived peptides. II. Influence of adrenocorticotropic hormone on the rise in intracellular free calcium concentration after T cell activation. Brain Behav Immun.

[16] Gatti G, Masera RG, Pallavicini L, Sartori ML, Staurenghi A, Orlandi F (1993). Interplay in vitro between ACTH, beta-endorphin, and glucocorticoids in the modulation of spontaneous and lymphokine-inducible human natural killer (NK) cell activity. Brain Behav Immun..

[17] Aebischer I, Stämpfli MR, Zürcher A, Miescher S, Urwyler A, Frey B (1994). Neuropeptides are potent modulators of human in vitro immunoglobulin E synthesis. Eur J Immunol..

[18] Riniker B, Sieber P, Rittel W, Zuber H (1972). Revised amino-acid sequences for porcine and human adrenocorticotrophic hormone. Nat New Biol..

[19] Schenkel-Hulliger L, Maier R, Barthe PL, Desaulles PA, Jarret A, Riniker B (1974). Biological activity of synthetic human corticotropin with revised amino acid sequence. Acta Endocrinol..

[20] Fiechtner J, Montroy T (2014). Treatment of moderately to severely active systemic lupus erythematosus with adrenocorticotropic hormone: a single-site, open-label trial. Lupus..

[21] Decker DA, Grant C, Oh L, Becker P, Young D, Jordan S (2014). Immunomodulatory effects of H.P. Acthar gel on B cell development in the NZB/W F1 mouse model of systemic lupus erythematosus. Lupus.

[22] Hodgkin PD, Lee JH, Lyons AB (1996). B cell differentiation and isotype switching is related to division cycle number. J Exp Med..

[23] Stavnezer J, Guikema JEJ, Schrader CE (2008). Mechanism and regulation of class switch recombination. Annu Rev Immunol..

[24] Peled JU, Kuang FL, Iglesias-Ussel MD, Roa S, Kalis SL, Goodman MF (2008). The biochemistry of somatic hypermutation. Annu Rev Immunol..

[25] Arnason BG, Berkovich R, Catania A, Lisak RP, Zaidi M (2013). Mechanisms of action of adrenocorticotropic hormone and other melanocortins relevant to the clinical management of patients with multiple sclerosis. Mult Scler..

[26] Berkovich R, Agius MA (2014). Mechanisms of action of ACTH in the management of relapsing forms of multiple sclerosis. Ther Adv Neurol Disord..

[27] Cooper A, Robinson SJ, Pickard C, Jackson CL, Friedmann PS, Healy E (2005). Alpha-melanocyte-stimulating hormone suppresses antigen-induced lymphocyte proliferation in humans independently of melanocortin 1 receptor gene status. J Immunol..

[28] Benko AL, Olsen NJ, Kovacs WJ (2014). Glucocorticoid inhibition of activation-induced cytidine deaminase expression in human B lymphocytes. Mol Cell Endocrinol..

[29] Maaser C, Kannengiesser K, Specht C, Lügering A, Brzoska T, Luger TA (2006). Crucial role of the melanocortin receptor MC1R in experimental colitis. Gut..

[30] Kannengiesser K, Maaser C, Heidemann J, Luegering A, Ross M, Brzoska T (2008). Melanocortin-derived tripeptide KPV has anti-inflammatory potential in murine models of inflammatory bowel disease. Inflamm Bowel Dis..

[31] Lee DJ, Biros DJ, Taylor AW (2009). Injection of an alpha-melanocyte stimulating hormone expression plasmid is effective in suppressing experimental autoimmune uveitis. Int Immunopharmacol..

[32] Edling AE, Gomes D, Weeden T, Dzuris J, Stefano J, Pan C (2011). Immunosuppressive activity of a novel peptide analog of α-melanocyte stimulating hormone (α-MSH) in experimental autoimmune uveitis. J Neuroimmunol..

[33] Cusick MF, Libbey JE, Oh L, Jordan S, Fujinami RS (2014). Acthar gel treatment suppresses acute exacerbations in a murine model of relapsing-remitting multiple sclerosis. Autoimmunity..

[34] Botte DAC, Noronha IL, Malheiros DMAC, Peixoto TV, de Mello SBV (2014). Alpha-melanocyte stimulating hormone ameliorates disease activity in an induced murine lupus-like model. Clin Exp Immunol..

[35] Spies CM, Straub RH, Cutolo M, Buttgereit F (2014). Circadian rhythms in rheumatology—a glucocorticoid perspective. Arthritis Res Ther..

[36] Tzioufas AG, Tsonis J, Moutsopoulos HM (2008). Neuroendocrine dysfunction in Sjogren's syndrome. Neuroimmunomodulation..

[37] Quax RA, Manenschijn L, Koper JW, Hazes JM, Lamberts SWJ, van Rossum EFC (2013). Glucocorticoid sensitivity in health and disease. Nat Rev Endocrinol..

